# Incidence, Spread and Mechanisms of Pyrethroid Resistance in European Populations of the Cabbage Stem Flea Beetle, *Psylliodes chrysocephala* L. (Coleoptera: Chrysomelidae)

**DOI:** 10.1371/journal.pone.0146045

**Published:** 2015-12-30

**Authors:** Dorte H. Højland, Ralf Nauen, Stephen P. Foster, Martin S. Williamson, Michael Kristensen

**Affiliations:** 1 Department of Agroecology, Aarhus University, Forsøgsvej 1, 4200 Slagelse, Denmark; 2 Bayer CropScience AG, Pest Control Biology, Alfred Nobel Str. 50, 40789 Monheim, Germany; 3 Department of Biological Chemistry and Crop Protection, Rothamsted Research, West Common, Harpenden AL5 2JQ, United Kingdom; Institute of Zoology, Chinese Academy of Sciences, CHINA

## Abstract

**Background:**

Cabbage stem flea beetle (CSFB), *Psylliodes chrysocephala* L. (Coleoptera: Chrysomelidae) is a major early season pest of oilseed rape throughout Europe. Pyrethroids have been used for controlling this pest by foliar application, but in recent years control failures have occurred, particularly in Germany due to the evolution of knock-down resistance (kdr). The purpose of this study was to investigate the incidence and spread of pyrethroid resistance in CSFB collected in Germany, Denmark and the United Kingdom during 2014. The level of pyrethroid resistance was measured in adult vial tests and linked to the presence of *kdr* genotypes.

**Results:**

Although *kdr* (L1014F) genotypes are present in all three countries, marked differences in pyrethroid efficacy were found in adult vial tests. Whereas Danish CSFB samples were in general susceptible to recommended label rates, those collected in the UK mostly resist such rates to some extent. Moderately resistant and susceptible samples were found in Germany. Interestingly, some of the resistant samples from the UK did not carry the *kdr* allele, which is in contrast to German CSFB. Pre-treatment with PBO, prior to exposure to λ-cyhalothrin suggested involvement of metabolic resistance in UK samples.

**Conclusion:**

Danish samples were mostly susceptible with very low resistance ratios, while most other samples showed reduced sensitivity in varying degrees. Likewise, there was a clear difference in the presence of the *kdr* mutation between the three countries. In the UK, the presence of *kdr* genotypes did not always correlate well with resistant phenotypes. This appears to be primarily conferred by a yet undisclosed, metabolic-based mechanism. Nevertheless our survey disclosed an alarming trend concerning the incidence and spread of CSFB resistance to pyrethroids, which is likely to have negative impacts on oilseed production in affected regions due to the lack of alternative modes of action for resistance management purposes.

## Introduction

The Brassicaceae family of crops is of agricultural importance worldwide due to their nutritional, medical and crop rotation potential, making them of significant economic value [1]. Some of the more important species of this family are oilseed rape (*Brassica napus*), cabbage, cauliflower, broccoli and various leafy vegetables, which all have a high nutritional value. Oilseed rape has been cultivated for many years in Europe, with more than 100,000 ha in Denmark, 564,000 ha in the UK and 1,400,000 ha in Germany, which is likely to increase in the future [2].

Oilseed rape has a range of pests, which destroy the pods or the stem, causing reduced yields and therefore reduced profit for growers. Some of the most important pests of this crop include pollen beetle (*Meligethes aeneus*), cabbage stem flea beetle (CSFB) (*Psylliodes chrysocephala*) and weevil species (*Ceutorhynchus* spp.). In several countries CSFB are the second most important pest after pollen beetles [3]. They are the first insect pest to infest winter oilseed rape in the UK [4], and have been an issue for many years in that country [5] and others such as Germany [3]. Like pollen beetles, CSFB also lives on turnip, mustard and cabbage [1] and produces one generation per year [6]. Adults emerge from summer diapause in mid to late August where they migrate to winter rape crops. On winter rape, eggs are laid normally during the autumn, but oviposition can continue into winter and the following spring if temperatures are high enough. The eggs are laid in cracks in the soil close to the base of the plant or in the lower part of the plant itself [7]. Females can produce up to 1,000 eggs during their life-span, making it a fast reproducing pest [6]. The rhythm of oviposition is dependent on temperature (4–12°C), and can modify whether a female lays her eggs all at once, in one raft, or in smaller rafts slightly apart in time [6].

The main damage caused by oilseed rape pests is due to tunneling of feeding larvae, [7] that move from ageing to healthy tissue, which makes plants more vulnerable to damage under certain weather conditions. Adult flea beetles feed heavily on young seedlings causing an inhibition of plant growth. It is important to monitor the crop during this time since an infestation can become serious in a few days [8].

Several control methods have been used for oilseed rape protection including insecticide application and push-pull strategies[9]. The large area of European soil used for growing winter oilseed rape makes pest control an essential part of production. In recent years, CSFB control has primarily relied on seed treatment with systemic neonicotinoid insecticides such as clothianidin, imidacloprid and thiamethoxam. If needed, spraying with pyrethroids has proved necessary in autumn if neonicotinoid levels have worn off and the dressing no longer has an effect [3]. However, since December 2013 this is no longer an option due to a European regulatory restriction on neonicotinoid treatment of seeds brought in because of the alleged harmful effects of these compounds on bees [10]. As a result, the lack of alternative insecticides has added increased selection pressure on pyrethroids as control agents for CSFB, which has apparently driven the development of resistance. CSFBs are in the field throughout the year, so control of other pests also has an effect on CSFB populations. For example, when adult pollen beetles emerge and fly into the field in spring, chemical control with pyrethroids is initiated. Spraying of pyrethroids aimed at pollen beetle, seed pod weevils and Brassica pod midge can continue throughout the summer, which also potentially increases selection on CSFB. Therefore, control problems observed for pollen beetle might also apply for CSFB.

An important mechanism of pyrethroid resistance is conferred by target-site mutation in the voltage-gated sodium channel gene, known as knock-down resistance (kdr or super-kdr). The mutations result in insensitivity to pyrethroids. Several mutations have been reported, but the most common involves the substitution of a leucine at position 1014 to phenylalanine (L1014F). This mutation was initially discovered in houseflies, but has since been observed in many insect pest species [11]. Several studies have suggested reduced control efficacy of pollen beetles by pyrethroids, due to both target-site and metabolic resistance [12,13]. This constant exposure to pyrethroids has imposed strong pressures culminating in control failure against resistant individuals.

The presence of the *kdr* allele has also been shown in German CSFB populations. Recently, Zimmer *et al*. (2014) observed resistance associated specifically by this mutation [3]. However, the resulting change in sensitivity was not great enough to protect beetle populations from the full pyrethroid field rate; i.e., they had a susceptible phenotype at this dose, but failed to be controlled with lower doses suggesting an impact on residual activity. In other oilseed rape pests, such as pollen beetle, a metabolic detoxification mechanism, based on over expression of a P450, has been observed [14].

Understanding the molecular basis of resistance, including discovering the function of the genes responsible, is an important step for pest management strategies. Novel strategies and development of new insecticides are crucial for keeping pesticide resistance at a low frequency as well as maintaining the diversity of control measures [15]. Furthermore, effective monitoring can limit unnecessary use of pesticides. Over-use of pesticides reduces the economic competitiveness of the crop and threatens biodiversity [7] causing environmental problems.

Here, we report on the incidence and spread of pyrethroid resistance in CSFB populations from Germany, Denmark and the UK. Bioassays were performed at up to six doses of λ-cyhalothrin. The frequency of *kdr* (heterozygotes and homozygotes) in these samples was also determined and related to resistance phenotype.

## Materials and Methods

### Insects

The beetles were collected on private land with consent of the owner. The field collection did not involve endangered or protected species. Live adult CSFBs were collected from oilseed rape fields in Germany, Denmark and the UK in 2014. In total 41 samples were collected from Germany, nine samples from Denmark, and 30 samples from the UK (S1 Table). Insects were collected in perforated plastic bags or plastic containers with some oilseed rape plant material and tissue paper, transferred to the laboratory and kept at low temperature (ranging from 4–12°C) until bioassays were done. The beetles were allowed to equilibrate to room temperature prior to bioassay. Only live, mobile beetles were used for bioassay.

### Bioassay

The test method used is based on IRAC (Insecticide Resistance Action Committee) method 031, with additional insecticide concentrations [16]. Glass vials were coated on the inner surface with different concentrations of λ-cyhalothrin (Table 1) dissolved in acetone as described by Zimmer *et al* (2014) [3]. Different doses, ranging from 0.16% to 100% of the recommended field application rate of λ-cyhalothrin, were used (2–4 replicates per concentration). Two UK samples were also exposed to 133% field rate. Glass vials treated with acetone alone served as controls. After 24 h, the number of cabbage stem flea beetles severely affected (dead or moribund) were scored and results expressed in percentage mortality. Beetles that were capable of coordinated movement were scored as ‘mobile/unaffected’. Some beetles from the UK were treated with PBO to assess metabolic resistance. After pre-exposure to PBO, beetles were bio-assayed alongside adults treated as normal by being moved to either untreated vials or vials coated with lambda-cyhalothrin at the 100% field dose.

**Table 1:** Bioassay doses for cabbage stem flea beetles. The λ-cyhalothrin doses used in this study expressed in ng per cm^2^ and % of field application.

ng cm^-2^ (g per ha)	% of field application
0.12 (0.012)	0.16
0.6 (0.06)	0.8
3 (0.3)	4
15 (1.5)	20
75 (7.5)	100
100 (10.0)	133

### DNA purification

Extraction of gDNA from Danish beetles was performed according to the manufacturer’s protocol for the DNeasy Blood and Tissue Kit (Qiagen). Gel electrophoresis and spectrophotometry (Nanodrop) were performed to assess the integrity and the concentration of each DNA sample.

Preparation of gDNA from German populations were performed according to the manufacturer’s protocol for the blackPREP Tick DNA/RNA Kit. Genomic DNA was extracted from UK samples using DNAzol reagent (Life Technologies) according to the supplier's recommended protocol. DNA from each beetle was dissolved in a final volume of 40μL and 1.5μL aliquots taken for *kdr* genotyping by TaqMan assay.

### Detection of *kdr* alleles

The presence of the L1014F *kdr* mutation in the German samples was detected by pyrosequencing as described by Zimmer *et al*. (2014) [3]. These samples were tested at Bayer CropScience, Monheim, Germany. UK and Danish beetles were tested for the same mutation by TaqMan assay, a method that uses fluorescent dye labelled oligonucleotide probes for allele-selective amplification and detection of wild-type and/or mutant gene fragments in a modified real-time PCR assay [17]. Primer and probe sequences for the assay were designed against gene sequences flanking the *kdr* mutation using Primer Express software v.2.0 (Life Technologies). Primers csfb_kdr_F (GGACTGTATGCTAGTCGGTGATGT) and csfb_kdr_R (GCAAAGCCAAGAAGAGATTCAGTA) are standard oligonucleotides with no modification. The probe csfb_kdr_V (TTACCACAAGATTACC) was labelled with VIC at the 5’ end for the detection of the wild-type allele, and the probe csfb_kdr_M (TTACCACAAAATTACC) was labelled with 6-FAM for detection of the resistant allele. Each probe also had a 3' non-fluorescent quencher and a minor groove binder at the 3' end. PCR assay reactions (15 μL) contained 1.5 μL of genomic DNA, 7.5 μL of SensiMix Probe mix (Bioline Reagents Ltd), 800 nM of each primer and 200 nM of each probe. Reactions were run on an ABI 7900HT (Applied Biosystems) using temperature cycling conditions of 10 min at 95°C, followed by 40 cycles of 95°C for 10 s and 60°C for 45 s. The increase in VIC and 6-FAM fluorescence was monitored in real time by acquiring each cycle on the yellow channel (530 nm excitation and 555 nm emission) and green channel (470 nm excitation and 510 emission) of the 7900HT, respectively.

## Results

**Fig 1:**
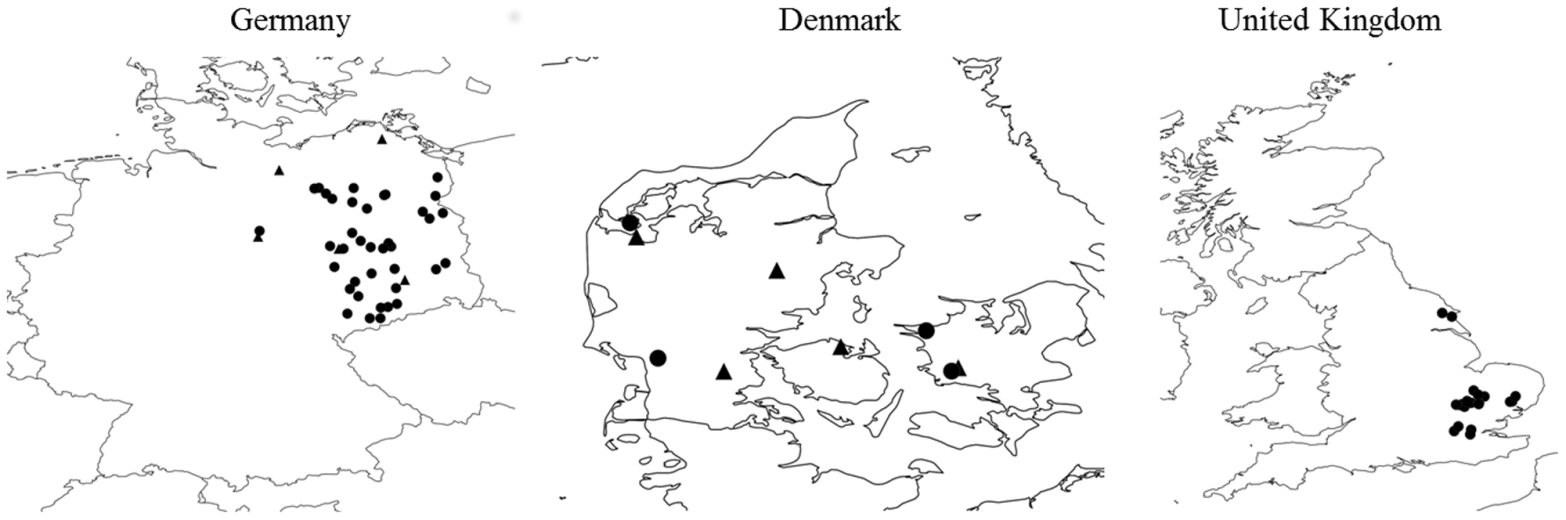
Locations of cabbage stem flea beetle samples. The triangles indicate samples where a full dose-response and *kdr* frequency was obtained.

CSFB samples were obtained from different locations in Germany, Denmark and the UK (Fig 1). We intended to assess whether the resistance observed previously in samples from Northern Germany could have spread to Denmark as well as other regions of Germany. We also investigated whether pyrethroid control failures, first reported in 2013, in the UK were due to resistance.

### Resistance status in Denmark

**Fig 2:**
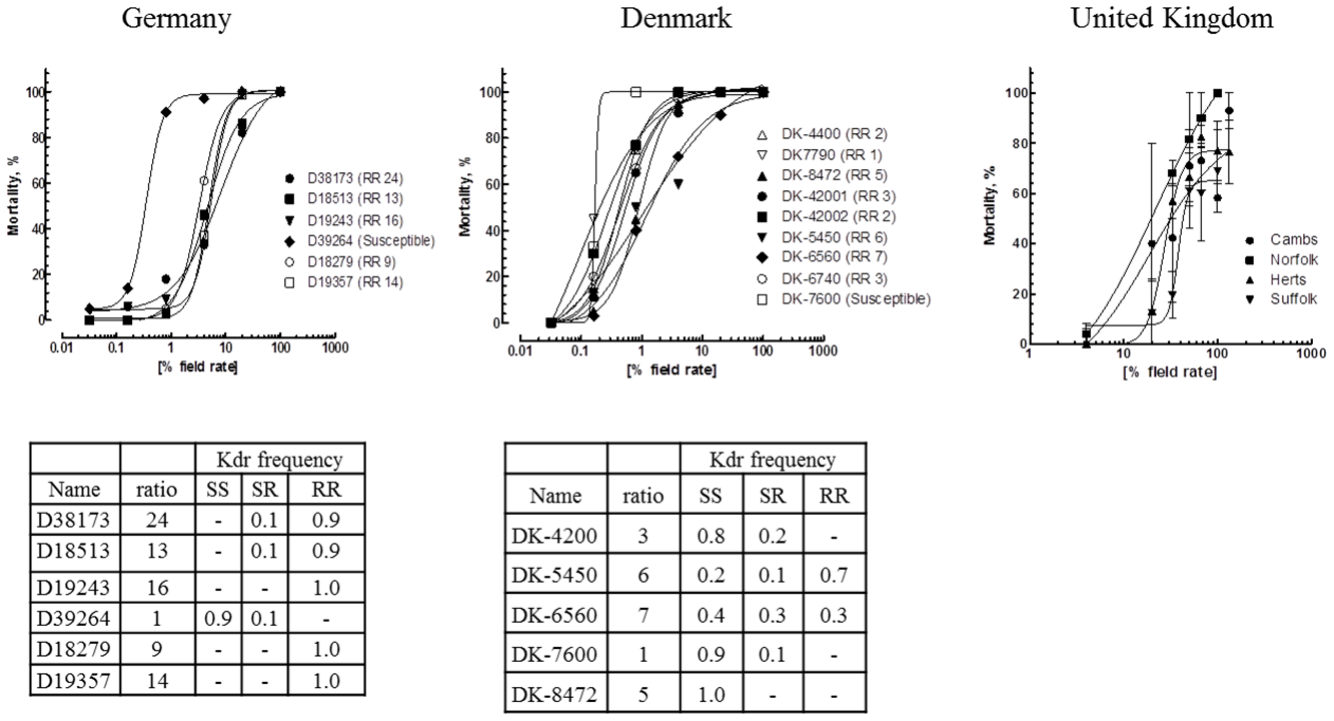
Dose-response curves and frequency of the kdr mutation for each of the three countries of this study. It was not possible to calculate resistance ratios (RR) for the UK samples, as they are much less sensitive. Six representative samples were analyzed from Germany, five from Denmark and four from the UK.

The Danish samples were collected from three main parts of Denmark, representing the entire country (Fig 1). Most of the samples were found to show 100% mortality at 20% of the field rate (0.015 μg cm^-2^), but mortality figures well below 100% at 4% of the field rate, indicating resistance when compared to fully susceptible samples, which are supposed to show 90% mortality at 0.8% of the field rate (e.g. D39364 from Germany). One sample (DK-6560) showed slightly higher resistance, with 20% of the label rate killing 93% of the beetles. In general, treatment with 100% of the recommended field rate caused 100% mortality, whereas 4% caused mortalities of >90% in seven of nine cases. Two samples (DK-6560 and DK-5450) had mortalities below 90% at 4% of the recommended label rate, with 72% and 60%, respectively (Fig 2). A total of 51 representative beetles from five Danish samples were tested for the *kdr* allele which proved to be present, in either heterozygotes or homozygotes, in four samples (Table 2, Fig 2). Samples from the west (DK-7600) and east (DK-8472) of Jutland (the peninsula north of Germany) showed no *kdr* (RR) homozygotes, with only one heterozygote (SR) beetle being found (Table 2, Fig 2). However, homozygote *kdr* beetles were found in the south of Jutland with five (26.5%) of the 19 beetles tested from this region being RR. The sample from Funen (DK-5450) had 11 homozygotes (68.8%) and 2 heterozygotes 2 (12%) out of a total of 16 beetles. The two samples showing homozygote *kdr* alleles were also those with the highest resistance ratio (at LC_50_), of 7 and 6, respectively (Fig 2).

**Table 2:** Frequencies of the kdr mutation. Populations tested.

	Region	Populations	N^a^	*kdr* status			
				SS	SR	RR	%RR
Germany	LS	1	12	9	0	3	25
	SAC	12	85	85	0	0	0
	SAH	3	35	14	12	9	26
	BRB	25	237	131	25	81	34
	Total	41	369	239(64.8%)	37(10.0%)	93(25.2%)	
Denmark	WZ	3	6	5	1	-	0
	FYN	1	16	3	2	11	68.8
	SJ	2	19	8	6	5	26.2
	WJ	2	9	8	1	-	0
	EJ	1	1	1	-	-	0
	Total	9	51	25 (49.0%)	10(19.6%)	16(31.4%)	
England	HFS	8	176	58	40	78	44.3
	CBS	8	73	19	14	40	54.8
	SF	8	121	28	22	71	58.7
	NF	4	51	31	6	14	27.5
	YS	3	58	0	0	58	100
	BFS	2	0	0	0	0	0
	ES	2	15	6	1	8	53.3
	Total	35	494	142(28.7%)	83(16.8%)	269(54.5%)	

^a^ Beetles tested for *kdr*

LS: Lower Saxony, SAC: Saxony, SAH: Saxony-Anhalt, BRB: Brandenburg, WS: Western Zealand, FYN: Funen, SJ: Southern Jutland, WJ: Western Jutland, EJ: Eastern Jutland, HFS: Hertfordshire, CBS: Cambridgeshire, SF: Suffolk, NF: Norfolk, YS: Yorkshire, BFS: Bedfordshire, ES: Essex.

### Resistance status in Germany

In total 41 German samples were collected from four regions, primarily from the north-east of the country (Fig 1). Bioassays performed on a few selected samples showed resistant beetles, with resistance ratios ranging from 9 to 24 based on log-dose probit-mortality data when compared to a susceptible strain. However, treatment with 100% of the recommended λ-cyhalothrin label rate still resulted in close to 100% mortality (Fig 2). In the susceptible sample, 10% were *kdr* heterozygotes and 90% homozygotes susceptible (SS) for the L1014 allele, resulting in approx. 90% mortality at 0.8% of the field rate. In the remaining five samples homozygote *kdr* (RR) individuals prevailed (Fig 2).

Beetles representing the 41 samples (N = 369) were tested for their *kdr* genotype. All beetles tested from the region of Saxony in 2014 were susceptible homozygotes (SS). In the other three regions, susceptible homozygotes (SS) were also prevalent (Table 2). Samples from Saxony-Anhalt had frequencies of homozygote resistant individuals of 17%-36%, while the single sample from Lower Saxony had 25% RR individuals. CFSB samples from Brandenburg showed the most diverse *kdr* profile of the four regions, with some samples solely containing *kdr* susceptible (SS) beetles and some samples containing beetles with resistant alleles. An overall fraction of almost 65% of the beetles tested for the *kdr* mutation were homozygotes (SS) for the susceptible allele, while 25% were homozygotes (RR) for the resistant allele (Table 2). Approximately 50% of the samples tested in Germany were either susceptible or resistant homozygotes while the remaining half of the samples was a mix of *kdr* genotypes.

### Resistance status in the UK

In the UK, 30 CSFB samples were collected from seven different counties, primarily in the south-eastern part of England (Fig 1). Beetles were tested primarily to measure their response at the recommended λ-cyhalothrin field dose rate (and a few other concentrations) to advise growers if resistant beetles were present in their oilseed rape crop. Pyrethroid resistance was detected in 20 samples, with mobile beetles being seen after 24h exposure to 100% of the recommended label rate of λ-cyhalothrin (7.5 g ai per ha). There was a suggestion that samples not containing mobile (resistant) beetles were located further north. Dose response curves for the UK samples are shown in Fig 2 for four counties, where there was sufficient data to analyse in this way.

Table 2 shows the frequency of the *kdr* genotypes in the UK CSFB samples collected from the seven counties. All three genotypes were present in the UK population in 2014 with *kdr*-RR (homozygotes) prevailing in all counties except Norfolk. Interestingly, all of the beetles in the three samples from Yorkshire were *kdr* homozygotes (RR). However, none of these proved to be resistant to the full λ-cyhalothrin field rate. Furthermore, *kdr* susceptible homozygotes (SS), not carrying the *kdr* target site mutation, were not restricted to the ‘affected’ or ‘dead beetle’ phenotypes seen at the 100% λ-cyhalothrin field dose rate, i.e. they were also found in beetles that were scored as mobile (resistant). For example, in the Cambridgeshire, Hertfordshire and Suffolk samples SS genotypes represented 18.2%, 34.8% and 80% of the mobile beetles. This suggests that *kdr* was not the only resistance mechanism present in CSFB in the UK in 2014. To investigate this further, additional bioassays were done on two samples (collected from Bedfordshire and Hertfordshire) where beetles were pre-treated in coated vials for 1 h with the xenobiotic metabolism inhibitor, piperonyl butoxide (PBO), at a dose of 0.011 mg cm^-2^, a compound used to test for the presence of metabolic-based resistance. The beetles were then bio-assayed alongside adults treated as normal by being moved to either untreated vials or vials coated with λ-cyhalothrin at the 100% field dose. The resulting data are shown in Table 3. PBO alone had little effect on adults with 100% (Bedfordshire) and 87.5% (Hertfordshire) mobile beetles in those moved to untreated vials. Respectively, there were also 40% and 46.7% mobile beetles present when exposed to λ-cyhalothrin alone at the field rate. However, all adults that were pre-treated with PBO prior to exposure to λ-cyhalothrin at the 100% field rate were killed. This is strong evidence that resistant beetles carry a metabolic-based mechanism which confers a significant part of resistance to λ-cyhalothrin. The identity of this mechanism needs to be investigated further to establish which particular enzyme, such as a P450, is involved.

**Table 3:** Effect of pre-treatment with the synergist PBO in two UK cabbage stem flea beetle samples.

Sample	Treatment	N^a^	resistant beetles (%)	kdr genotype (%)		
				SS	SR	RR
Hertfordshire	Untreated	9	89	22	33	45
	PBO	8	88	38	24	38
	100% λ-cyhalothrin	15	47	47	13	40
	PBO + 100% λ-cyhalothrin	11	27	27	27	46
Bedfordshire	Untreated	10	100	30	30	40
	PBO	8	100	25	13	62
	100% λ-cyhalothrin	15	40	27	27	46
	PBO + 100% λ-cyhalothrin	15	0	33	20	47

^a^Total number of beetles genotyped.

## Discussion

A topical, coated glass vial bioassay method was used for quickly testing CSFB adults for resistance to pyrethroids. This was used to test German, Danish and UK CSFB samples collected in 2014. A recent study on CSFB sampled in northern Germany revealed the presence of pyrethroid resistance, particularly in north-western Mecklenburg-Western Pomerania at different collection sites [18]. The calculated resistance ratios based on adult vial tests were up to 81-fold and the authors observed a significant decline in pyrethroid susceptibility between the years 2007 and 2011. A later study conducted by Zimmer *et al*. [3] revealed that some of the German samples collected in 2010 carry the well-known L1014F *kdr* mutation known to confer pyrethroid target-site resistance in several different pests [19], including some known to feed on oilseed rape such as pollen beetle [13] Here we investigated for the first time several UK and Danish CSFB populations for pyrethroid resistance and additionally analysed them for the presence of *kdr*. Furthermore we extended the study to a larger number of collection sites in Germany, i.e. other federal states than Mecklenburg-Western Pomerania, in order to investigate the geographic spread of *kdr*-based pyrethroid resistance in Germany.

In the UK, most of the samples contained mobile (resistant) beetles after exposure to doses equivalent to the field rate (7.5 g a.i. per ha) or above (10 g a.i. per ha) of λ-cyhalothrin. This demonstrated widespread, strong resistance in this pest in this country in 2014. The *kdr* resistance mutation (L1014F), known to confer pyrethroid resistance in other pests such as aphids and pollen beetles, was found in both the heterozygous and homozygous form. However, resistance in the bioassays done on the UK samples did not compare well with *kdr* genotype, as *kdr*-susceptible (SS) adults were sometimes scored as being fully mobile after being treated with the full field rate of λ-cyhalothrin. Conversely, all beetles collected from Yorkshire were *kdr* homozygotes (RR) but still showed susceptibility to λ-cyhalothrin applied at the field rate. These observations suggest that CSFB in this country were carrying at least one other form of resistance, a hypothesis that was verified in bioassays pre-treating beetles with PBO, which circumvents the presence of metabolic-based resistance. Further research is needed to identify the metabolic-based mechanism found in UK CSFBs which could be based on the over-expression of a P450, as seen in pollen beetles [12]. In the meantime, using *kdr* frequencies alone will not accurately describe pyrethroid resistance in this pest in this country. Therefore, bioassays on live beetles will continue to be needed to diagnose resistance phenotype in the UK where control failures with pyrethroids have coincided with an EU-led restriction that imposed the loss of neonicotinoid seed treatments on oilseed rape from December 2013. This situation highlights the importance of maintaining a range of compounds for controlling oilseed rape pests including flea beetles and aphids.

Most samples from Denmark proved to be more susceptible than those from the UK and Germany. In one sample, obtained from the south of Jutland, mobile beetles were present after exposure to 20% λ-cyhalothrin for 24 hours. However, there were no survivors at 100% of the recommended label rate, classifying this specific sample as having decreased susceptibility towards λ-cyhalothrin. The other eight Danish samples showed 100% mortality at 20% λ-cyhalothrin categorizing them as susceptible according to the IRAC method 031. However, a fully susceptible population is one, where 90% mortality is achieved with 0.8% of the field rate, which was the case for the DK-7660 sample only. For the moment this suggests limited resistance problems in CSFBs in Denmark which is supported by no reports of pyrethroid control failures. However, to avoid development of resistance in the future, measures can be made, such as seed treatment with appropriate insecticides with a different mode of action from the sodium channel. Alternating between different modes of action decreases the selection pressure for specific insecticide, delaying resistance development.

A number of Danish beetles were assessed for the presence of the *kdr* allele. Wild-type L1014 homozygote allele were observed for 10 of the 30 beetles surviving 4% and 15 out of 18 beetles surviving 0.8% of the label rate. Heterozygotes (SR) were observed in individuals surviving 4%, as well as in individuals not surviving 0.8% of the recommended field rate. Individuals that were homozygous for the *kdr* mutation (RR) were identified in two of the samples tested, representing 15 individuals surviving 4% and 20% of the recommended field rate as well as a single beetle, not surviving 0.8%. Beetles (all from one sample) surviving 20% of the recommended field rate were all homozygotes for the *kdr* mutation.

Field resistance to λ-cyhalothrin seems not to be prevalent in Danish beetles since most beetles died at 20% of the label rate, but the presence of the single sample, close to Germany, and one from Funen with *kdr* homozygote resistant individuals could indicate the selection for higher target-site resistance frequency. However, one must keep in mind that other resistance mechanism may be present. In general, the frequencies of the resistant allele ranged from 0.06 to 0.75 in the Danish samples, which further indicates the pre-stages of resistance. It seems the levels of *kdr* presence in Denmark and their relationship to resistance correspond to that of Germany, where resistance ratios ranged from 8–20-fold for the analysed German samples. However, all beetles surviving 20% field rate proved to be *kdr* homozygotes, indicating a role of *kdr* in survival at relatively low doses of λ-cyhalothrin. Our results suggest the presence of target-site resistance with no indication of control failure. However, one must keep in mind that bioassay data cannot directly be translated to the field. The low levels of λ-cyhalothrin resistance in Danish CSFBs show that pyrethroids are currently still a possible control agent against this pest in Denmark. However, no alternatives currently exists, so the low levels of resistance reported here might not stay low for long.

The analyses of the German samples for the presence of *kdr* suggest a spread of the resistance allele further south, as we were able to detect RR homozygotes in both Brandenburg and Saxony-Anhalt. The resistance allele was not found in any CSFB samples collected in 2014 in Saxony, located south of the two federal states mentioned above. Six German sampling sites provided enough individuals for a more detailed dose-response analyses including *kdr* genotyping (Fig 2). Interestingly only one of the samples (D39264) turned out to be highly susceptible to pyrethroids, i.e. showing 90% mortality at 0.8% of the recommended field rate. This sample originates from Saxony and included a single *kdr* heterozygote and nine SS homozygotes. However its high sensitivity also suggests the absence of other (metabolic) resistance mechanisms as shown for some of the UK samples, which were much more resistant in the absence of the *kdr* allele. The other German samples originating from Lower Saxony, Saxony-Anhalt and Brandenburg were collected from pyrethroid treated oilseed rape fields. These included 90–100% RR *kdr* homozygote, which is reflected by a decent number of survivors at 20% of the field rate. The resistance ratios are between 9- and 24-fold, thus indeed suggesting a fairly low impact of the L1014F mutation on resistance levels, provided that metabolic resistance mechanisms are lacking. Principally the data obtained from the German samples showed a correlation between resistant beetles and prevalence of the *kdr* mutation, indicating the importance of *kdr* in regions of lower pyrethroid efficacy in Germany. The susceptible beetles (D39264) were collected from fields with no control failure while—as mentioned above—beetles mostly homozygous for the *kdr* resistance allele were from fields where control failures had been reported. In Germany it was also recently shown that the presence of *kdr* contributes to pyrethroid resistance [3] leading to control failure in the field and a lack of residual activity. Thus the samples analysed in 2014 confirmed the emerging trend towards more resistant CSFB populations observed earlier [3,18].

There was a good correlation between mobility/survival of CSFBs at high concentrations of λ-cyhalothrin and the presence of the *kdr* allele, especially in Germany. However, in the UK, *kdr* seems not to be main contributor to control failures, with metabolic resistance obviously playing a significant role as synergist trials revealed. Control failure of CSFB seems to become a major issue in Germany and England, but is not yet observed to a similar extent in Denmark. However, λ-cyhalothrin remains an efficient control agent in Denmark, despite a single sample showing decreased susceptibility. But this might not be for long, due to lack of alternative control agents. Monitoring of resistance levels of Danish samples is essential to the continued use of λ-cyhalothrin in Denmark.

Due to the lack of any alternative modes of action and the relatively low impact of *kdr* on pyrethroid field efficacy against CSFB, it is for the time being tempting to continue to rely on pyrethroids, but such a strategy is for sure not sustainable and could result in a collapse of oilseed rape production in those regions where CSFB outbreaks occur. In some regions in Germany up to three pyrethroid applications were necessary in autumn 2014 for CSFB control, compared to only one application in former years. Recently neonicotinoid seed treatments provided a second mode of action for resistance management purposes in terms of early season protection of young seedlings from flea beetle attack. Their recent EU wide ban as oilseed rape seed treatment is likely to have strong implications for oilseed rape production and without any doubt will increase pyrethroid selection pressure, and possibly facilitating the emergence of additional resistance mechanisms, which are likely to have the potential to resist current label rates as already observed in some spots in the UK. Worth to mention here that due to increasing CSFB pressure regulatory authorities in several EU member states, including Denmark, UK, Finland and Estonia provided derogations for neonicotinoid seed treatment in oilseed rape in 2015.

## Supporting Information

S1 Table Cabbage stem flea beetle collection sites. (PDF)
