# Supplementary material for: Incidence, Spread and Mechanisms of Pyrethroid Resistance in European Populations of the Cabbage Stem Flea Beetle, Psylliodes chrysocephala L. (Coleoptera: Chrysomelidae)
Source: PLoS One. 2015 Dec 30;10(12):e0146045. doi: 10.1371/journal.pone.0146045 (PMC4696833; doi:10.1371/journal.pone.0146045)
Supplement: S1 Table — (PDF) [file pone.0146045.s001.pdf]

S1 Table. Cabbage stem flea beetle collection sites

Country	Town	Region	Latitude, longitude
United Kingdom	Impington	Cambrigdeshire	52.1505, 0.0707
	Great Wilbraham	Cambrigdeshire	52.11, 0.16
	Great Wilbraham	Cambrigdeshire	52.11, 0.16
	Tadlow. Royston	Cambrigdeshire	52.06, -0.075
	Cambridge	Cambrigdeshire	52.121, 0.073
	Arrington	Cambrigdeshire	52.081, -0.035
	Stowbridge	Norfolk	52.38, 0215
	Thetford	Norfolk	52.25, 0.451
	Feltwell	Norfolk	52.29, 0.311
	Pulman Market	Norfolk	52.254, 1.135
	Baldock	Hertfordshire	51.592, -0.122
	Rothamsted	Hertfordshire	51.491, -0.2132
	Rothamsted	Hertfordshire	51.491, -0.2132
	Rothamsted	Hertfordshire	51.491, -0.2132
	Rothamsted	Hertfordshire	51.491, -0.2132
	Rothamsted	Hertfordshire	51.491, -0.2132
	Harpenden	Hertfordshire	51.491, -0.2132
	Royston	Hertfordshire	52.025, 0.013
	Sandy	Bedfordshire	52.07, -0.17
	Ousden	Suffolk	52.12, 0.33
	Wickhambrook	Suffolk	52.1, 0.334
	Barrow	Suffolk	52.15, 0.35
	Stradishall	Suffolk	52.083, 0.326
	Wickhambrook	Suffolk	52.1, 0.334
	Brockford	Suffolk	52.141, 1.061
	Gipping	Suffolk	52.135, 1.016
	Ryton. North	Yorkshire	54.08, -0.475
	Driffield	Yorkshire	54.002, -0.264
	Driffield	Yorkshire	54.002, -0.264
	Takely	Essex	51.52, 0.16
	Ongar	Essex	51.42, 0.14
Germany	Almosen	Brandenburg	51.580632, 14.097578
	Betzin	Brandenburg	52.757901, 12.754676
	Blüthen	Brandenburg	53.160787, 11.820965
	Crussow	Brandenburg	53.004635, 14.085720
	Feldheim	Brandenburg	52.010504, 12.826792
	Freudenberg	Brandenburg	52.698314, 13.838791
	Hohenstein	Brandenburg	52.564926, 13.970083
	Jänickendorf	Brandenburg	52.021308, 13.225201
	Karstädt	Brandenburg	53.147022, 11.733419

	Klein Oßning	Brandenburg	51.700117, 14.280310
	Kleinow	Brandenburg	53.049494, 11.959157
	Lüderdorf	Brandenburg	52.088029, 13.173197
	Neutrebbin	Brandenburg	52.672207, 14.229262
	Preußnitz	Brandenburg	52.131977, 12.631910
	Prignitz	Brandenburg	52.948181, 12.079353
	Rauschendorf	Brandenburg	53.032046, 13.114880
	Sonnenberg	Brandenburg	53.018255, 13.089571
	Trampe	Brandenburg	53.364981, 14.125967
	Wittstock	Brandenburg	53.156776, 12.495747
	Wollin	Brandenburg	52.287249, 12.466865
	Wusterhausen	Brandenburg	52.882813, 12.469684
	Göttingen	Lower Saxony	52.326216, 10.667977
	Althen	Saxony	51.339873, 12.522527
	Audenhain	Saxony	51.499635, 12.844084
	Breitenau	Saxony	50.849864, 13.162031
	Euba	Saxony	50.836285, 13.024021
	Freiberg	Saxony	50.910024, 13.338593
	Greifenhain	Saxony	51.058090, 12.586855
	Großzossen	Saxony	51.199023, 12.423263
	Neundorf	Saxony	50.632207, 13.015913
	Paltzschen	Saxony	51.216510, 13.319307
	Werdau	Saxony	50.720720, 12.374731
	Übigau	Saxony	51.589066, 13.292262
	Zwönitz	Saxony	50.631217, 12.809578
	Buhlendorf	Saxony-anhalt	52.031208, 12.037942
	Ragösen	Saxony-anhalt	51.980919, 12.298703
	Zörbick	Saxony-anhalt	51.626804, 12.122311
Denmark	Fuglebjerg	West Zealand	55.355897, 11.450414
	Flakkebjerg	West Zealand	55.325056, 11.390832
	Kalundborg	West Zealand	55.686731, 11.163374
	Egense	Funen	55.545003, 10.398003
	Sommersted	South Jutland	55.323670, 9.350937
	Bramming	South Jutland	55.441006, 8.761026
	Holstebro	West Jutland	56.527179, 8.567961
	Thyholm	West Jutland	56.652657, 8.511435
	Aarhus	East Jutland	56.228209, 9.825486
